# Fluorodeoxyglucose Metabolic Phenotypes Across Molecular Subgroups of Primary Colorectal Cancer: A Retrospective Three-Group Analysis

**DOI:** 10.3390/jcm15187113

**Published:** 2026-09-14

**Authors:** Riku Morita, Takashi Norikane, Yuka Yamamoto, Yuri Manabe, Mitsumasa Murao, Takafumi Obata, Katsuya Mitamura, Hiroyuki Okuyama, Yoshihiro Nishiyama

**Affiliations:** 1 Department of Radiology, Faculty of Medicine, Kagawa University, Miki-cho, Kita-gun 761-0793, Japan; morita.riku@kagawa-u.ac.jp (R.M.); manabe.yuri@kagawa-u.ac.jp (Y.M.); mitamura.katsuya@kagawa-u.ac.jp (K.M.);; 2 Department of Medical Oncology, Faculty of Medicine, Kagawa University, Miki-cho, Kita-gun 761-0793, Japan; okuyama.hiroyuki@kagawa-u.ac.jp

**Keywords:** colorectal cancer, FDG PET/CT, *RAS*, *BRAF*, mucinous component

## Abstract

**Background/Objectives:** Increased FDG uptake has been reported in *RAS*-mutant colorectal cancer (CRC), whereas the FDG metabolic phenotype of *BRAF*-mutant CRC is less well characterized. We compared FDG PET-derived parameters among *RAS*-mutant, *BRAF*-mutant, and *RAS*/*BRAF* double-wild-type primary CRCs and explored associations between mucinous components and FDG uptake within each subgroup. **Methods:** This retrospective study included 105 patients with 106 treatment-naive primary CRCs (44 *RAS*-mutant, 11 *BRAF*-mutant, and 51 double-wild-type tumors). Maximum standardized uptake value (SUVmax), peak standardized uptake value (SUVpeak), mean standardized uptake value (SUVmean), metabolic tumor volume (MTV), and total lesion glycolysis (TLG) were measured. Mucinous components were assessed from original histopathological reports in 86 evaluable tumors. **Results:** SUVmax, SUVpeak, SUVmean, and TLG differed among the three subgroups (*p* = 0.006, 0.015, 0.009, and 0.039, respectively), whereas MTV did not (*p* = 0.130). After Bonferroni correction, *RAS*-mutant tumors had higher SUVmax, SUVpeak, and SUVmean than double-wild-type tumors. No significant pairwise differences involving *BRAF*-mutant tumors were observed. Mucinous components were present in 11/34 *RAS*-mutant, 4/10 *BRAF*-mutant, and 4/42 double-wild-type tumors (*p* = 0.015). In exploratory within-subgroup analyses, *BRAF*-mutant tumors with mucinous components had lower SUVmax, SUVpeak, and SUVmean than those without mucinous components (exact *p* = 0.010 for each; Bonferroni-adjusted *p* = 0.048 for each); no corresponding differences were observed in the other subgroups. **Conclusions:** *RAS*-mutant primary CRC showed higher metabolic intensity than *RAS*/*BRAF* double-wild-type CRC, whereas *BRAF*-mutant tumors did not differ significantly from either subgroup. The association between mucinous components and lower metabolic intensity in *BRAF*-mutant CRC should be considered exploratory and hypothesis-generating.

## 1. Introduction

Colorectal cancer (CRC) remains a major cause of cancer morbidity and mortality and is biologically heterogeneous [1]. Specific molecular alterations influence prognosis and treatment selection. For example, mutations in *RAS* genes (*KRAS* and *NRAS*) predict resistance to anti-epidermal growth factor receptor therapy [2], while the *BRAF* V600E mutation is a therapeutic target in advanced disease [3]. *BRAF*-mutant CRC has distinctive clinicopathological features, including an aggressive clinical course, frequent proximal location, mucinous differentiation, and microsatellite instability-high (MSI-high) status [4,5].

Fluorodeoxyglucose positron emission tomography/computed tomography (FDG PET/CT) provides quantitative information on tumor glucose metabolism. Maximum standardized uptake value (SUVmax), peak standardized uptake value (SUVpeak), and mean standardized uptake value (SUVmean) reflect complementary aspects of metabolic intensity, whereas metabolic tumor volume (MTV) and total lesion glycolysis (TLG) quantify metabolically active volume and total glycolytic burden [6,7]. Associations between *KRAS* or *RAS* mutations and increased FDG uptake in CRC have been examined in clinical studies, meta-analyses, radiomics studies, and intratumoral heterogeneity analyses [8,9,10,11,12]. However, further insight may be gained by comparing FDG metabolic phenotypes across distinct molecular subgroups rather than focusing solely on increased FDG uptake in *RAS*-mutant CRC.

The metabolic phenotype of *BRAF*-mutant CRC, a molecular subgroup associated with adverse clinical outcomes [13], remains less well characterized. A previous study combined *KRAS*- and *BRAF*-mutant tumors into a single mutation-positive group, which may obscure metabolic differences between these molecular subgroups [8]. Moreover, mucin-rich tumors can demonstrate reduced FDG accumulation, an imaging finding that has been attributed to abundant extracellular mucin and lower viable cellular density [14]. However, the extent to which mucinous histology is associated with variation in FDG uptake within *BRAF*-mutant CRC remains unclear.

The present study therefore compared FDG PET-derived parameters among treatment-naive *RAS*-mutant, *BRAF*-mutant, and *RAS*/*BRAF* double-wild-type primary CRCs. We also conducted exploratory within-subgroup analyses to examine the association between the presence of mucinous components and FDG uptake in each molecular subgroup.

## 2. Materials and Methods

### 2.1. Patients

This retrospective study enrolled patients with newly diagnosed, histologically confirmed adenocarcinoma of the colon or rectum who underwent pretreatment FDG PET/CT and molecular analysis for *RAS* and *BRAF* mutations between June 2020 and May 2026. Examinations performed in patients with a blood glucose concentration > 150 mg/dL, measured before FDG administration on the day of PET/CT, were excluded. Patients who had received surgery, chemotherapy, radiotherapy, or other antitumor treatment before PET/CT were also excluded.

The final cohort comprised 105 patients with 106 primary CRCs. One patient had two synchronous primary tumors in the sigmoid colon and rectum. Both tumors underwent separate PET measurements and were treated as distinct tumor-level observations; all other patients contributed one primary tumor.

The study was initially approved by the institutional review board of our university on 5 September 2025, with subsequent amendments approved on 29 July 2026, and 7 August 2026. The study was conducted in accordance with the Declaration of Helsinki and relevant national ethical guidelines. The requirement for informed consent was waived because of the retrospective design.

### 2.2. FDG PET/CT Acquisition and Image Analysis

FDG was produced using an automated synthesis system coupled to an HM-18 cyclotron (QUPID; Sumitomo Heavy Industries Ltd., Tokyo, Japan) under standardized radiopharmaceutical production conditions. PET/CT was conducted using the Biograph Vision 600 system (Siemens Medical Solutions USA Inc., Knoxville, TN, USA). Patients fasted for at least 5 h before intravenous FDG administration (3.7 MBq/kg).

Whole-body PET acquisition was performed from the mid-skull to the upper thigh after a 60 min uptake period, with an acquisition time of 2 min per bed position. A low-dose, non-contrast CT scan covering the same field of view was obtained for attenuation correction and anatomical localization. PET data were reconstructed using ordered-subset expectation maximization with point-spread-function and time-of-flight modeling (three iterations and five subsets).

Quantitative PET analysis was performed using syngo.via (Siemens Medical Solutions USA Inc., Knoxville, TN, USA) by an experienced nuclear medicine physician blinded to mutation status. SUVmax, SUVpeak, SUVmean, MTV, and TLG were measured for each primary tumor. MTV was defined as the volume of voxels demonstrating uptake of at least 40% of the tumor SUVmax. SUVmean was calculated within the MTV-defined volume, and TLG was calculated as SUVmean multiplied by MTV.

### 2.3. Molecular and Histopathological Assessment

*RAS* and *BRAF* mutation analyses were performed using pretreatment tumor tissue specimens. The MEBGEN *RAS*KET-B kit (Medical & Biological Laboratories Co., Ltd., Nagoya, Japan) was used, employing multiplex polymerase chain reaction–reverse sequence-specific oligonucleotide analysis with fluorescent bead-based detection using the Luminex system [15]. The assay detects hotspot variants in *KRAS* and *NRAS* exons 2, 3, and 4 and the *BRAF* V600E mutation in exon 15.

Tumors were classified as *RAS*-mutant, *BRAF*-mutant, or *RAS*/*BRAF* double-wild-type. None of the tumors had concurrent *RAS* and *BRAF* mutations. The presence of a mucinous component was determined retrospectively from the original histopathological report and was recorded when mucinous histology was explicitly described. As the absence of a mucinous component could not be reliably established from limited non-surgical samples, this analysis was restricted to 86 tumors with a specific histological subtype documented in the pathology data. Cases recorded as non-surgical were excluded. No centralized pathological re-review was performed, and the proportion of the mucinous component was not quantitatively assessed. MSI status was categorized as MSI-high or -low when available. Six tumors with untested status were excluded from the MSI-specific analyses.

### 2.4. Statistical Analysis

Continuous variables are presented as medians with interquartile ranges unless otherwise indicated. Categorical variables are presented as numbers and percentages of tumors. Primary analyses were performed at the tumor level. FDG PET-derived parameters were compared among the three molecular subgroups using the Kruskal–Wallis test. When the overall test was significant, post hoc pairwise comparisons were performed with Bonferroni adjustment. Categorical variables were compared using Fisher’s exact test or the Fisher–Freeman–Halton exact test, as appropriate. Because one patient contributed two synchronous primary tumors, sensitivity analyses of the molecular-subgroup comparisons were performed by retaining only one of the two tumors from this patient, using each tumor in turn.

Exploratory analyses of mucinous components were conducted in histologically evaluable tumors. Within each molecular subgroup, PET parameters were compared between tumors with and without a mucinous component using the Mann–Whitney U test. Exact *p* values were calculated, and a Bonferroni correction was applied across the five PET parameters within each molecular subgroup. These within-subgroup analyses were exploratory and were not used to formally test whether the magnitude of the association with mucinous components differed among the molecular subgroups. Finally, MSI-high and -low tumors were compared using the Mann–Whitney U test.

Statistical analyses were performed using IBM SPSS Statistics, version 30.0 (IBM Corp., Armonk, NY, USA) and EZR (Saitama Medical Center, Jichi Medical University, Saitama, Japan), a graphical user interface for R statistical software (R version 4.3.1) [16]. All tests were two-sided, and *p* values < 0.05 were considered significant.

## 3. Results

### 3.1. Patient and Tumor Characteristics

The cohort comprised 105 patients (57 men and 48 women; mean age, 70.9 years; range, 34–95 years) with 106 primary tumors. Molecular classification identified 44 *RAS*-mutant tumors, 11 *BRAF*-mutant tumors, and 51 *RAS*/*BRAF* double-wild-type tumors. Baseline characteristics are summarized in Table 1. Age and right-sided location did not differ among molecular subgroups. Sex distribution differed among groups (*p* = 0.018), and clinical stage distribution showed a borderline group difference (*p* = 0.049).

Among the 86 tumors evaluable for detailed histological components, a mucinous component was documented in 11 of 34 *RAS*-mutant tumors (32%), 4 of 10 *BRAF*-mutant tumors (40%), and 4 of 42 double-wild-type tumors (10%) (*p* = 0.015). MSI status was available for 100 tumors. MSI-high status was present in 1 of 41 *RAS*-mutant tumors (2%), 5 of 11 *BRAF*-mutant tumors (45%), and 1 of 48 double-wild-type tumors (2%) (*p* < 0.001).

### 3.2. FDG PET Parameters According to Molecular Subgroup

**Figure 1:**
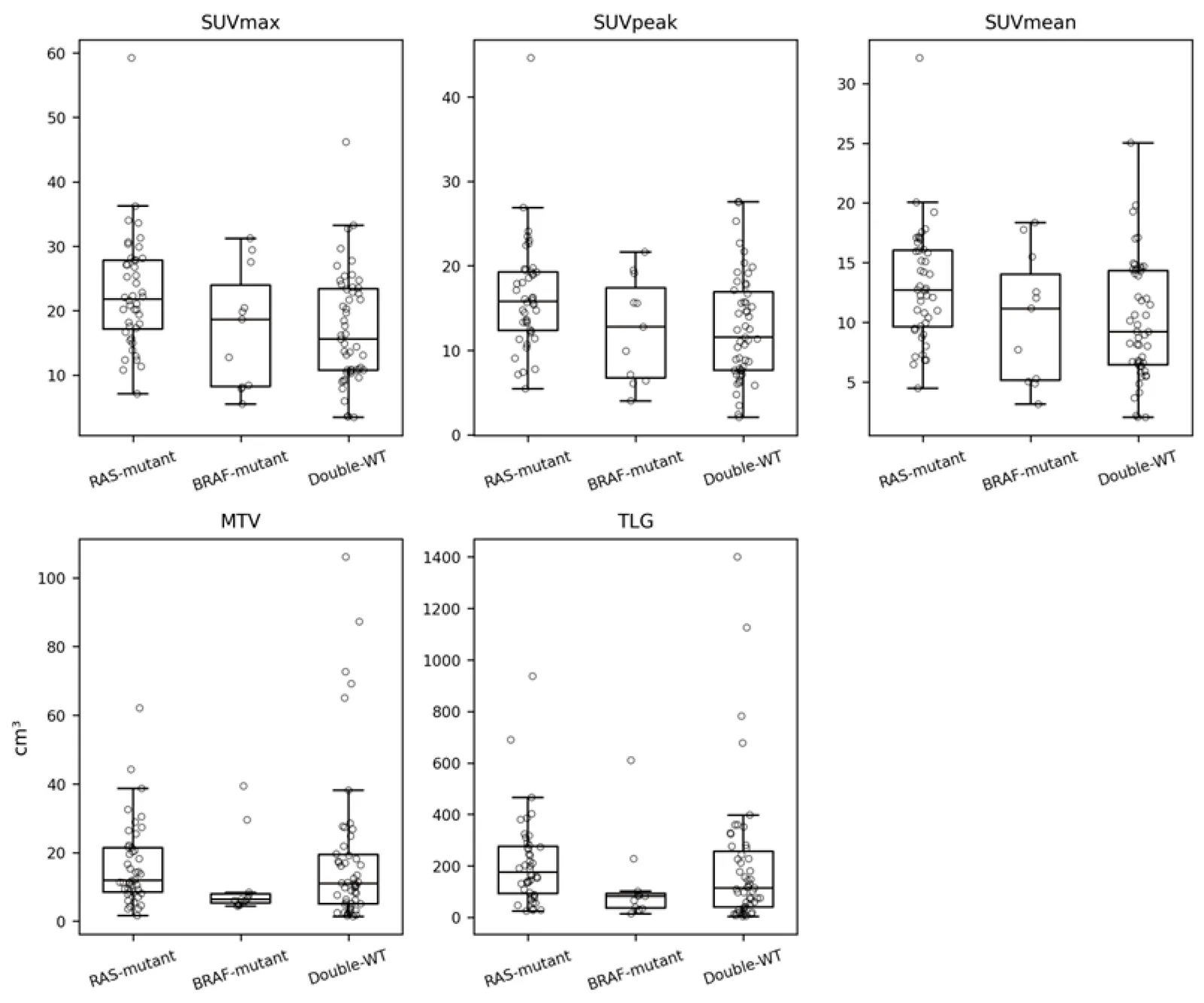
FDG PET-derived parameters according to molecular subgroup. Box-and-whisker plots with individual tumor observations show SUVmax, SUVpeak, SUVmean, MTV, and TLG in *RAS*-mutant, *BRAF*-mutant, and *RAS*/*BRAF* double-wild-type colorectal cancer. The boxes represent the interquartile range, and the horizontal line represents the median.

The distributions of FDG PET-derived parameters are shown in Figure 1 and summarized in Table 2. SUVmax, SUVpeak, SUVmean, and TLG differed significantly among the three molecular subgroups (*p* = 0.006, 0.015, 0.009, and 0.039, respectively), whereas MTV did not (*p* = 0.130). After Bonferroni correction, *RAS*-mutant tumors had higher SUVmax, SUVpeak, and SUVmean than double-wild-type tumors (adjusted *p* = 0.007, 0.017, and 0.008, respectively). No significant pairwise differences were observed between *RAS*-mutant and *BRAF*-mutant tumors or between *BRAF*-mutant and double-wild-type tumors. Although the overall difference in TLG was significant, none of the pairwise comparisons remained significant after correction.

In sensitivity analyses retaining only one of the two synchronous *RAS*-mutant tumors, the overall *p* values after exclusion of the sigmoid tumor were 0.007 for SUVmax, 0.020 for SUVpeak, 0.010 for SUVmean, 0.157 for MTV, and 0.052 for TLG; after exclusion of the rectal tumor, the corresponding *p* values were 0.004, 0.010, 0.006, 0.096, and 0.026, respectively. In both analyses, the *RAS*-mutant versus double-wild-type post hoc comparisons for SUVmax, SUVpeak, and SUVmean remained significant after correction, whereas no corrected pairwise TLG comparison was significant. The exploratory *BRAF*-mutant/mucinous-component comparison was unchanged because neither synchronous tumor belonged to the *BRAF*-mutant subgroup.

### 3.3. Exploratory Analysis of Mucinous Components and FDG Uptake

**Figure 2:**
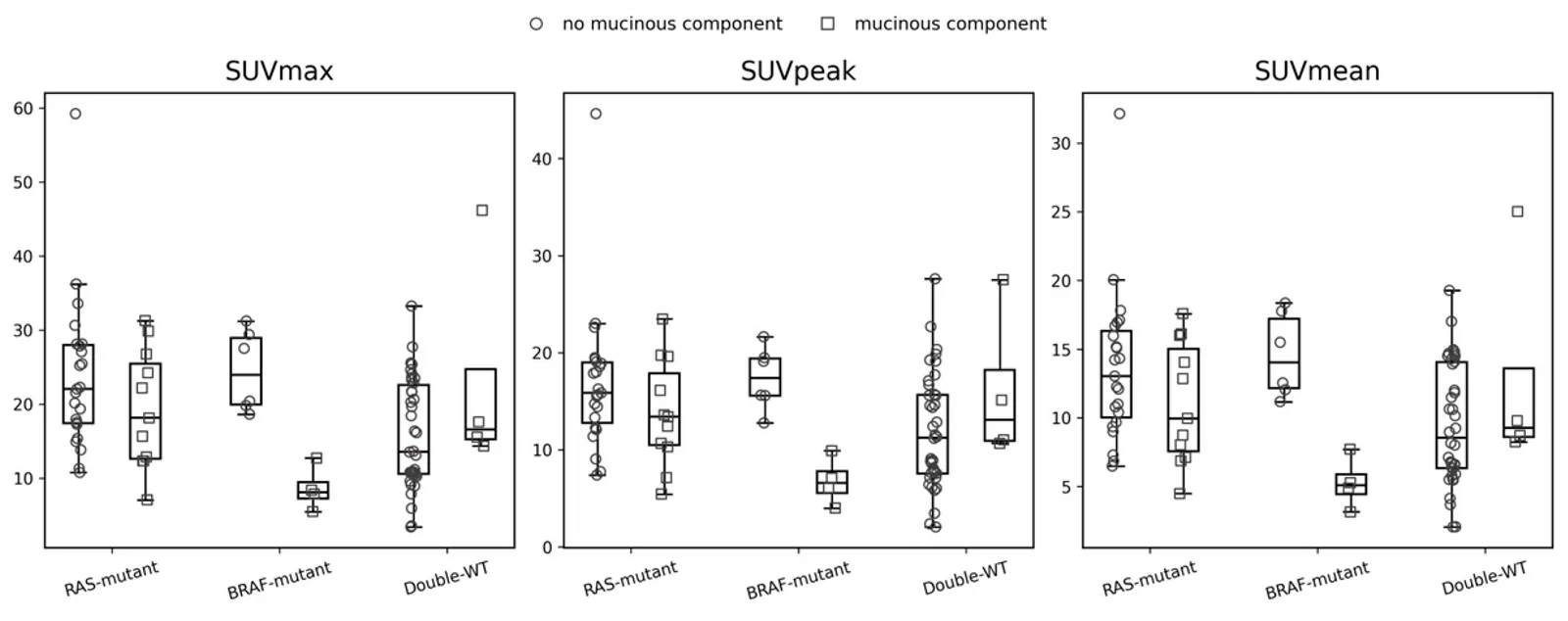
FDG PET-derived metabolic intensity according to mucinous component status within each molecular subgroup. Individual observations and box-and-whisker plots show SUVmax, SUVpeak, and SUVmean in tumors with and without a mucinous component. The comparisons are exploratory; in particular, the *BRAF*-mutant strata comprised only four tumors with and six tumors without a documented mucinous component and should be interpreted cautiously.

Exploratory within-subgroup comparisons according to the presence or absence of a mucinous component are shown in Figure 2 and Table 3. These analyses were performed separately within each molecular subgroup and should be interpreted as hypothesis-generating rather than as a formal demonstration that the association with mucinous components differs among molecular subgroups.

Within the *BRAF*-mutant subgroup, the four tumors with a mucinous component had lower SUVmax, SUVpeak, and SUVmean than the six tumors without a mucinous component. Median SUVmax was 8.14 versus 23.97, median SUVpeak was 6.59 versus 17.37, and median SUVmean was 5.08 versus 14.02 (exact *p* = 0.010 for each; Bonferroni-adjusted *p* = 0.048 for each). MTV and TLG did not differ significantly. No significant association between a mucinous component and any PET parameter was observed within the *RAS*-mutant or double-wild-type subgroups. Because these comparisons were performed separately within each molecular subgroup, they do not establish a subgroup-by-mucinous-component interaction.

### 3.4. MSI Status and FDG PET Parameters

In the 100 tumors with known MSI status, MSI-high tumors had numerically lower median SUVmax, SUVpeak, and SUVmean and a higher median MTV than MSI-low tumors. However, none of the five PET parameters differed significantly by MSI status (all *p* > 0.30). Similarly, within the *BRAF*-mutant subgroup, PET parameters did not differ significantly between MSI-high and -low tumors. Only seven MSI-high tumors were available overall, including five in the *BRAF*-mutant group; therefore, the absence of statistical significance should not be interpreted as excluding a potential association between MSI status and FDG uptake.

## 4. Discussion

The principal findings of the present study were that *RAS*-mutant tumors showed higher SUVmax, SUVpeak, and SUVmean than *RAS*/*BRAF* double-wild-type tumors, whereas *BRAF*-mutant tumors showed no significant differences from either of the other molecular subgroups. Thus, separation of *RAS*- and *BRAF*-mutant CRCs provided a more specific description of FDG metabolic phenotypes across molecular subgroups. In exploratory analyses of histologically evaluable tumors, the presence of a mucinous component was associated with lower SUVmax, SUVpeak, and SUVmean within the small *BRAF*-mutant subgroup. Given that this comparison involved only four tumors with and six tumors without a mucinous component, the finding should be regarded as exploratory and hypothesis-generating.

The higher SUVmax, SUVpeak, and SUVmean observed in *RAS*-mutant tumors than in double-wild-type tumors are consistent with previous studies reporting an association between *KRAS*/*RAS* mutations and increased FDG uptake in CRC [8,11,12]. Experimental work in colorectal cancer cells has shown that mutated *KRAS* can increase FDG accumulation through upregulation of glucose transporter 1 [17]. The present findings therefore support previous observations of a *RAS*-associated high-intensity metabolic phenotype while placing them in a three-group analysis that separately characterizes *BRAF*-mutant tumors.

Previous FDG PET studies examining molecular alterations in CRC have focused predominantly on *KRAS* or *RAS* mutations. A previous study combined *KRAS*- and *BRAF*-mutant tumors into a single mutation-positive group [8]. Such grouping may obscure mutation-specific metabolic patterns because the more prevalent *RAS*-mutant tumors can dominate the characteristics of the combined mutation-positive group. Dedicated data on the FDG metabolic phenotype of *BRAF*-mutant primary CRC therefore remain limited.

In the present study, *BRAF*-mutant tumors did not show uniformly low or high FDG uptake. The *BRAF*-mutant group did not differ significantly from either the *RAS*-mutant or double-wild-type group in pairwise comparisons of SUVmax, SUVpeak, SUVmean, MTV, or TLG, and both low- and high-uptake tumors were observed. These findings suggest heterogeneity in FDG uptake among BRAF-mutant tumors in this cohort and support analyzing *BRAF*-mutant tumors separately rather than assuming a common mutation-positive FDG phenotype with *RAS*-mutant tumors.

An exploratory finding was the association between mucinous components and FDG uptake. Mucinous components were evaluated in all three molecular subgroups, not only in *BRAF*-mutant tumors. Within the *BRAF*-mutant subgroup, tumors with a documented mucinous component had lower SUVmax, SUVpeak, and SUVmean than those without a mucinous component, whereas no significant within-subgroup differences were detected in *RAS*-mutant or double-wild-type tumors. However, these were separate exploratory comparisons, and the very small *BRAF*-mutant strata do not establish that the mucinous–FDG relationship is unique to *BRAF*-mutant CRC. Rather, the results generate the hypothesis that histological composition may contribute to variation in FDG uptake within *BRAF*-mutant tumors. Reduced FDG uptake in mucin-rich tumors is biologically plausible and has been attributed to abundant extracellular mucin and relatively low viable cellular density [14]. Nevertheless, the present study did not directly quantify mucin content, tumor cellular density, glucose transporter expression, glycolytic activity, or other molecular mechanisms. Accordingly, this proposed explanation should be regarded as a biologically plausible interpretation rather than a mechanism demonstrated by the present data.

MSI-high status was strongly enriched in the *BRAF*-mutant group, in which it was present in 45% of tumors compared with 2% in each of the other molecular groups, and *BRAF*-mutant tumors also more frequently contained mucinous components. Although no statistically significant differences in conventional FDG PET parameters were detected between MSI-high and MSI-low tumors, only seven MSI-high tumors were available overall. Therefore, the nonsignificant MSI analysis cannot exclude a potential effect of MSI on FDG uptake. Because *BRAF* mutation, MSI-high status, and mucinous morphology are biologically interrelated, their independent contributions could not be disentangled in this cohort, and confounding by MSI remains possible.

This study has several important limitations. First, it was retrospective and conducted at a single center. Second, only 11 *BRAF*-mutant tumors were included, and the key exploratory mucinous-component comparison was based on four tumors with and six tumors without a documented mucinous component. These subgroup findings are therefore vulnerable to small-sample instability and should be considered hypothesis-generating. Third, mucinous components were classified retrospectively from the original histopathological reports without centralized pathological re-review or quantitative assessment of the proportion of mucin, creating potential misclassification bias and preventing evaluation of a quantitative relationship between mucin content and FDG uptake. Fourth, MSI-high status was strongly enriched in the *BRAF*-mutant group, but the total number of MSI-high tumors was small; consequently, confounding by MSI could not be excluded. Fifth, one patient contributed two synchronous *RAS*-mutant tumors to the tumor-level analyses. Sensitivity analyses retaining either tumor confirmed the robustness of the SUVmax, SUVpeak, and SUVmean findings, although the overall TLG *p* value varied, indicating some sensitivity of this secondary parameter to the independence assumption. Sixth, the biological mechanisms underlying the observed association between mucinous components and lower FDG uptake were not directly investigated; cellular density, glucose transporter expression, and glycolytic activity were not assessed.

Future studies should prioritize larger multicenter cohorts with adequate numbers of *BRAF*-mutant and MSI-high tumors, centralized pathological re-review, and quantitative assessment of mucinous components. Such studies could determine whether the exploratory association between mucinous morphology and FDG uptake persists after accounting for MSI status and other clinicopathological factors. Standardized PET acquisition and reconstruction would also facilitate external validation and comparison of quantitative FDG PET parameters across institutions.

## 5. Conclusions

*RAS*-mutant primary CRC showed higher SUVmax, SUVpeak, and SUVmean than *RAS*/*BRAF* double-wild-type CRC, whereas *BRAF*-mutant CRC did not differ significantly from either molecular subgroup. In exploratory within-subgroup analyses, *BRAF*-mutant tumors with a documented mucinous component had lower metabolic intensity than those without. Given the very small *BRAF*-mutant strata, retrospective pathological classification, and potential confounding by MSI, this observation should be regarded as exploratory and hypothesis-generating. Histological composition may contribute to variation in FDG uptake within *BRAF*-mutant CRC, but larger studies with standardized, quantitative pathological assessment are needed.

## Figures and Tables

**Table 1:** Patient and tumor characteristics according to molecular subgroup.

Characteristic	*RAS*-Mutant (n = 44 Tumors)	*BRAF*-Mutant (n = 11 Tumors)	Double-Wild-Type (n = 51 Tumors)	*p* Value
**Age, years**	74.5 (64.8–79.3)	75.0 (57.5–77.5)	72.0 (65.0–77.5)	0.658
**Female sex**	24/44 (55%)	8/11 (73%)	17/51 (33%)	0.018
**Right-sided tumor**	13/44 (30%)	6/11 (55%)	15/51 (29%)	0.280
**Clinical stage I–II**	10/44 (23%)	5/11 (45%)	26/51 (51%)	0.049
**Clinical stage III**	18/44 (41%)	3/11 (27%)	10/51 (20%)	
**Clinical stage IV**	16/44 (36%)	3/11 (27%)	15/51 (29%)	
Mucinous component *	11/34 (32%)	4/10 (40%)	4/42 (10%)	0.015
**MSI-high ^†^**	1/41 (2%)	5/11 (45%)	1/48 (2%)	<0.001

Data are presented as the median (interquartile range) or number/total (%). Analyses were performed at the tumor level; one female patient contributed two synchronous *RAS*-mutant primary tumors. * Mucinous components were assessed from the original histopathological reports in 86 histologically evaluable tumors. ^†^ Six tumors with untested MSI status were excluded. MSI, microsatellite instability.

**Table 2:** FDG PET-derived parameters according to molecular subgroup.

Parameter	*RAS*-Mutant (n = 44)	*BRAF*-Mutant (n = 11)	Double-Wild-Type (n = 51)	*p* Value
**SUVmax**	21.81 (17.13–27.83)	18.64 (8.25–23.97)	15.57 (10.77–23.41)	0.006
**SUVpeak**	15.77 (12.38–19.27)	12.77 (6.76–17.37)	11.53 (7.64–16.90)	0.015
**SUVmean**	12.71 (9.63–16.03)	11.17 (5.15–14.02)	9.22 (6.46–14.34)	0.009
**MTV (cm^3^)**	11.90 (8.48–21.48)	6.37 (5.34–8.04)	11.05 (5.09–19.39)	0.130
**TLG**	175.84 (93.72–275.00)	81.84 (36.14–93.58)	114.17 (39.39–255.97)	0.039

Data are presented as the median (interquartile range). MTV, metabolic tumor volume; TLG, total lesion glycolysis.

**Table 3:** Exploratory within-subgroup comparison of FDG PET parameters according to mucinous component status.

Molecular Subgroup	Parameter	Mucinous Component Present	Mucinous Component Absent	Exact *p* Value	Bonferroni-Adjusted *p*	n (Present/Absent)
***RAS*** **-Mutant**	SUVmax	18.16 (12.63–25.49)	22.05 (17.42–27.98)	0.308	1.000	11/23
	SUVpeak	13.41 (10.50–17.88)	15.86 (12.79–18.99)	0.344	1.000	
	SUVmean	9.95 (7.56–15.02)	13.03 (10.03–16.33)	0.228	1.000	
	MTV	11.82 (8.88–15.96)	11.22 (6.92–20.99)	0.942	1.000	
	TLG	144.25 (76.89–188.45)	162.61 (79.14–256.60)	0.513	1.000	
***BRAF*** **-Mutant**	SUVmax	8.14 (7.28–9.48)	23.97 (19.96–28.93)	0.010	0.048	4/6
	SUVpeak	6.59 (5.55–7.81)	17.37 (15.59–19.39)	0.010	0.048	
	SUVmean	5.08 (4.45–5.88)	14.02 (12.17–17.19)	0.010	0.048	
	MTV	6.21 (5.67–12.16)	6.27 (5.10–7.97)	1.000	1.000	
	TLG	31.52 (25.71–82.06)	85.08 (82.39–97.30)	0.171	0.857	
**Double-Wild-Type**	SUVmax	16.60 (15.27–24.76)	13.59 (10.58–22.57)	0.327	1.000	4/38
	SUVpeak	13.08 (10.93–18.23)	11.26 (7.57–15.68)	0.349	1.000	
	SUVmean	9.25 (8.59–13.60)	8.56 (6.33–14.04)	0.394	1.000	
	MTV	19.22 (9.89–37.79)	9.34 (3.47–16.87)	0.199	0.997	
	TLG	190.32 (140.77–338.14)	75.76 (26.28–178.95)	0.132	0.660	

Data are presented as the median (interquartile range). Mucinous components were assessed from the original histopathological reports in 86 histologically evaluable tumors. These analyses were exploratory and do not constitute a formal test of a molecular-subgroup-by-mucinous-component interaction. MTV, metabolic tumor volume; TLG, total lesion glycolysis.

## Data Availability

The datasets generated and/or analyzed during the current study are available from the corresponding author upon reasonable request.

## Abbreviations

The following abbreviations are used in this manuscript:

CRC	Colorectal cancer
FDG PET/CT	Fluorodeoxyglucose positron emission tomography/computed tomography
MSI-high	Microsatellite instability-high
MTV	Metabolic tumor volume
SUVmax	Maximum standardized uptake value
SUVmean	Mean standardized uptake value
SUVpeak	Peak standardized uptake value
TLG	Total lesion glycolysis
